# Watercress (*Nasturtium officinale*) as a Functional Food for Non-Communicable Diseases Prevention and Management: A Narrative Review

**DOI:** 10.3390/life15071104

**Published:** 2025-07-15

**Authors:** Chikondi Maluwa, Blecious Zinan’dala, Hataichanok Chuljerm, Wason Parklak, Kanokwan Kulprachakarn

**Affiliations:** 1 School of Health Sciences Research, Research Institute for Health Sciences, Chiang Mai University, Chiang Mai 50200, Thailand; chikondi_maluwa@cmu.ac.th (C.M.); dysonblecious_zina@cmu.ac.th (B.Z.); hataichanok.ch@cmu.ac.th (H.C.); 2 Faculty of Clinical Sciences, Malawi College of Health Sciences, Blantyre Campus, Blantyre Private Bag 396, Malawi; 3 Faculty of Clinical Sciences, Malawi College of Health Sciences, Lilongwe Campus, Lilongwe P.O. Box 30368, Malawi; 4 Research Center for Non-infectious Diseases and Environmental Health, Research Institute for Health Sciences, Chiang Mai University, Chiang Mai 50200, Thailand; wason.p@cmu.ac.th

**Keywords:** watercress, non-communicable diseases (NCDs), phytochemicals, bioactive compounds, dietary interventions

## Abstract

Non-communicable diseases (NCDs) such as cardiovascular disease, diabetes, cancer, and chronic respiratory conditions are the leading causes of death globally, largely driven by modifiable lifestyle factors. With growing interest in dietary strategies for NCDs prevention and management, functional foods like watercress (*Nasturtium officinale*) have attracted attention for their rich phytochemical content and potential health benefits. This narrative review synthesizes 88 sources published between 2019 and March 2025, exploring the effects of watercress bioactive compounds on major NCDs. Watercress is abundant in glucosinolates, isothiocyanates (especially phenethyl isothiocyanate), flavonoids, vitamins, and minerals. These compounds contribute to antioxidant, anti-inflammatory, and metabolic regulatory effects. Preclinical and clinical studies show that watercress supplementation may improve lipid profiles, reduce oxidative stress, and modulate inflammation in cardiovascular and respiratory conditions. It also appears to enhance insulin function and reduce blood glucose levels. In cancer models, watercress extracts exhibit antiproliferative, pro-apoptotic, and chemoprotective properties, with selective toxicity towards cancer cells and protective effects on normal cells. These findings highlight the therapeutic potential of watercress as a dietary adjunct in NCDs prevention and management, supporting the need for further clinical research.

## 1. Introduction

Non-communicable diseases (NCDs), also known as chronic diseases, tend to be of long duration and are the result of a combination of genetic, physiological, environmental, and behavioral factors, accounting for 75% of non-pandemic-related deaths globally [1]. The main types of NCDs are cardiovascular diseases (including atherosclerosis, coronary heart diseases, and stroke), diabetes, cancers, and chronic respiratory diseases (including chronic obstructive pulmonary disease and asthma). These four groups of diseases account for 80% of all premature NCDs deaths [2]. Common risk factors, including tobacco use, inactivity, excessive alcohol consumption, unhealthy diets, and lack of physical exercise, contribute to the high mortality rate associated with NCDs [3]. As such, there is increasing interest in the role of dietary interventions in NCDs prevention and management. Functional foods, particularly those rich in phytochemicals, are gaining prominence in public health nutrition strategies.

The Brassicaceae family, often referred to as cruciferous vegetables like kale, cabbage, broccoli, watercress, and turnips, are famous for their impressive array of phytochemicals, especially glucosinolates, flavonoids, phenolic acids, and sulfur-containing compounds. These bioactive compounds are known for their strong antioxidant, anti-inflammatory, anticancer, and antidiabetic properties. For instance, glucosinolate derivatives like gluconasturtiin have shown great potential in preventing cancer and aiding detoxification. Plus, with the abundance of vitamins, carotenoids, and fiber, these vegetables further promote heart health, boost immune function, and help regulate metabolism [4,5,6,7,8].

Watercress (*Nasturtium officinale* W.T. Aiton), a nutrient-dense cruciferous vegetable and member of the Brassicaceae family which is found in most parts of Africa, Asia, Europe, and America, is an aquatic leafy green traditionally consumed for its peppery flavor and nutrient density. Beyond its culinary use, emerging research highlights its potential preventive and therapeutic benefits. Rich in glucosinolates, polyphenols, flavonoids, vitamins (A, C, E, B3, K), and minerals (calcium, iron, iodine, phosphorus, magnesium, and copper), watercress exhibits a range of bioactive properties that may mitigate the risk or progression of various chronic conditions [5,6,9,10,11]. Historically it has been used in traditional medicine in Europe, Asia, and Africa for its diuretic and detoxifying properties. Contemporary research highlights its potential in mitigating oxidative stress, inflammation, and carcinogenesis [12].

This narrative review explores the current evidence on the beneficial human health properties of watercress, with a focus on its effect on the following four major non-communicable diseases: cardiovascular diseases, diabetes, cancer, and chronic respiratory diseases.

## 2. Phytochemical Composition of Watercress

Watercress is recognized for its rich phytochemical content that contributes to its health-promoting properties. It contains high levels of glucosinolates, sulfur-containing compounds that are hydrolyzed into biologically active isothiocyanates, such as phenethyl isothiocyanate (PEITC) and methyl isothiocyanate (MEITC), which exhibit potential anticancer activities [7,9]. Additionally, watercress is a notable source of minerals including calcium, iron, iodine, phosphorus, magnesium, copper, and flavonoids, particularly quercetin, rutin, and kaempferol, which are compounds known for their antioxidant, anti-inflammatory, and cardioprotective effects [10,13,14].

The plant also provides carotenoids like lutein and beta-carotene, which play crucial roles in eye health and immune function [15]. Furthermore, vitamin C, an important antioxidant, is abundant in watercress, enhancing its ability to neutralize free radicals [16]. Additionally, presence of terpenes like α-terpinolene, limonene, caryophyllene oxide, and p-cymene-8-ol in watercress boast antioxidant, anti-inflammatory, and anticancer benefits while enhancing delightful aromas [4]. Minor constituents include phenolic acids such as caffeic acid and ferulic acid, contributing additional antioxidant potential [9] (Table 1).

## 3. Effects of Watercress on Major NCDs

**Figure 1:**
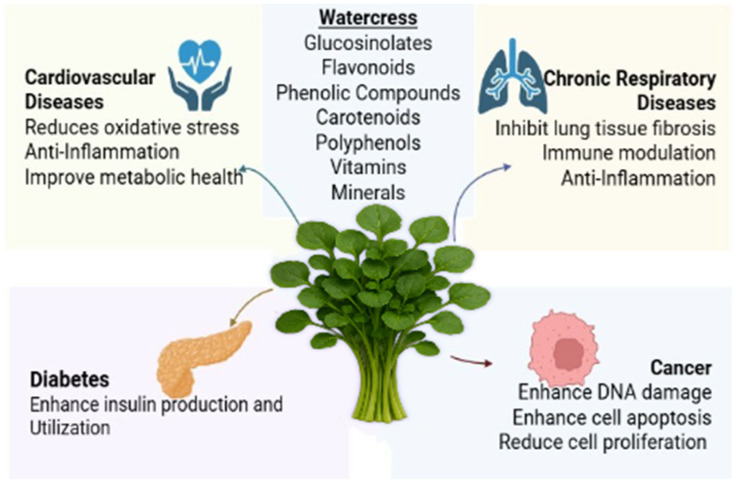
Effects of watercress in the prevention and management of NCDs.

Watercress has shown potential in mitigating NCDs due to its high nutrient density and bioactive compounds. Rich in antioxidants like vitamin C, beta-carotene, and glucosinolates, watercress may reduce oxidative stress and inflammation, key drivers of chronic conditions such as cardiovascular disease (CVD) and diabetes. It also inhibits lung tissue fibrosis in chronic respiratory diseases and enhances cell apoptosis in cancer conditions (Figure 1) [17,18,19,20,21]. Studies suggest that regular consumption can improve lipid profiles by lowering low-density lipoprotein (LDL) cholesterol and triglycerides, thereby reducing CVD risk. Furthermore, its high folate content supports metabolic health, potentially lowering homocysteine levels linked to stroke and hypertension [12,22,23,24,25].

Watercress may also play a role in cancer prevention, one of the major NCDs, due to its glucosinolate-derived compounds like PEITC, which exhibit anti-carcinogenic properties. Research indicates that PEITC can inhibit cancer cell proliferation and enhance cell apoptosis [6,13,26,27,28,29]. Furthermore, its low glycemic index and high fiber content aid in blood sugar regulation by increasing insulin production and utilization, benefiting individuals with diabetes [10,24,30,31,32,33]. Moreover, watercress may reduce chronic respiratory disease risks by lowering inflammation and oxidative stress in the lungs, hence inhibiting lung fibrosis [34,35,36,37,38,39].

Recent clinical trials focusing on the effects of watercress on human health have primarily utilized extracts from its aerial parts, particularly the leaves, stems, and flowers, which are packed with beneficial phytochemicals such as polyphenols, glucosinolates, and isothiocyanates. In studies by Clement et al. in 2020 and Clement et al. in 2021, participants received a standardized watercress extract (SENO) from leaves at a dosage of 750 mg/kg/day over a span of 5 weeks. This led to notable improvements, including reduced oxidative stress, lower levels of CRP, improved LDL cholesterol, and better creatinine levels among overweight and disabled individuals. Similarly, Sedaghattalab et al. in 2021 administered 500 mg/day of a hydroalcoholic extract (EENO) of watercress leaves to hemodialysis patients for 4 weeks, resulting in decreased malondialdehyde (MDA) and blood urea nitrogen (BUN), along with increased superoxide dismutase (SOD) activity and improved lipid profiles. Research by Schulze et al. in 2021 and Schuchardt et al. in 2019 demonstrated that consuming fresh watercress leaves had immediate antioxidant and anti-inflammatory effects, while also supporting immune function in healthy adults. In addition, Rashid et al. in 2021 discovered that watercress leaf extract helped lower blood glucose levels in diabetic patients. In another study, Shakerinasab et al. in 2024 provided asthma patients with 500 mg of watercress leaf extract twice daily for 4 weeks and observed a reduction in oxidative stress and an increase in antioxidant capacity. These findings indicate that both watercress extracts and fresh leaves may offer a wide range of benefits across various populations in the prevention and management of NCDs [24,25,36,40,41,42,43] (Table 2).

### 3.1. Effects of Watercress on Cardiovascular Diseases

**Figure 2:**
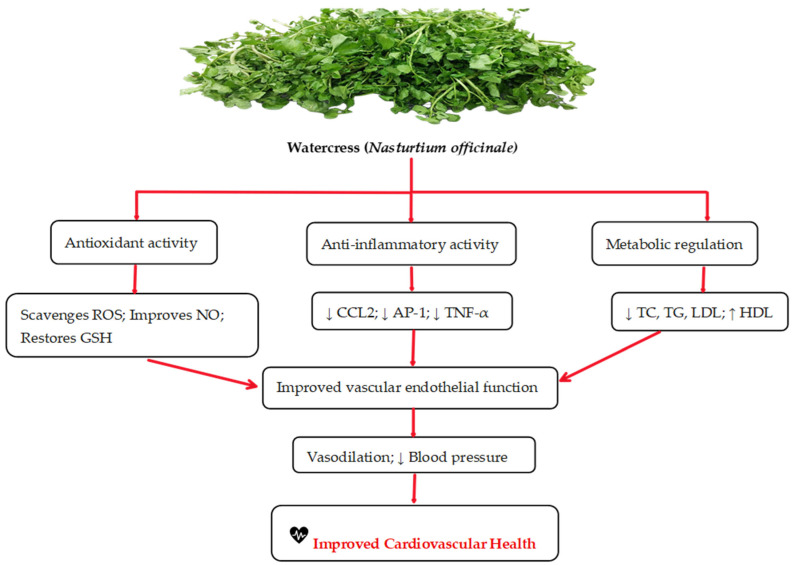
Mechanism of action of watercress in improving cardiovascular health. Note: ↓: decreased; ↑: increased; ROS: reactive oxygen species; NO: nitric oxide; GSH: glutathione; CCL2: chemokine (C-C motif) ligand 2; AP-1: activator protein 1; TNF-α: tumor necrosis factor-alpha; TC: total cholesterol; TG: triglyceride; LDL: low-density lipoprotein; HDL: high-density lipoprotein.

CVDs are a group of disorders that affect the heart and blood vessels. They include conditions such as coronary heart disease, hypertension, atherosclerosis, and heart failure, and collectively represent a major cause of global morbidity and mortality [44]. Pathways that lead to CVDs involve complex interactions between metabolic processes, oxidative stress, and inflammatory signaling [45,46] (Figure 2).

Metabolites such as bradykinin, des-Arg(9), nitric oxide (NO), and N-acetylglycine play key roles in regulating blood pressure and endothelial function. Unlike bradykinin which acts on B2 receptors, des-Arg(9) acts on B1 receptors, triggering vasodilation with a delayed and prolonged effect. Genetic variations in the pathways involving these metabolites have been linked to hypertension and atherosclerosis. Similarly, dimethylglycine and N-acetylmethionine are associated with increased cardiovascular disease risk, likely through mechanisms related to oxidative stress [47,48]. Proteins such as AP-1 and CCL2 contribute to vascular inflammation by promoting smooth muscle cell proliferation and recruiting monocytes. AP-1 plays a key role in driving vascular inflammation by increasing the expression of pro-inflammatory genes, such as cytokines and adhesion molecules. On the other hand, CCL2 works to recruit monocytes through binding to its receptor CCR2, which together enhances endothelial dysfunction, promotes leukocyte infiltration, and contributes to the development of atherosclerosis [49]. Additionally, the adaptor protein p66Shc exacerbates oxidative stress by increasing mitochondrial reactive oxygen species (ROS) production, thereby accelerating endothelial dysfunction and vascular remodeling [50,51]. Several metabolic pathways, including caffeine metabolism, bile acid biosynthesis, and branched-chain amino acid (BCAA) catabolism, intersect with CVD mechanisms. Altered BCAA levels are associated with insulin resistance and cardiac hypertrophy, while gut microbiota-derived metabolites such as trimethylamine N-oxide (TMAO) contribute to systemic inflammation and thrombosis. Furthermore, the WNT/β-catenin signaling pathway plays a crucial role in cardiac development and repair, with its dysregulation resulting into congenital heart defects and myocardial fibrosis [45,52,53].

Watercress has shown in animal and human models to have an effect on cardiovascular diseases through its antioxidant, anti-inflammatory, and metabolic modulation mechanisms [9,10,12,40]. In a 28-day animal study, Olufemi et al. explored the effects of bisphenol A (BPA), dibutyl phthalate (DBP), and their combination with or without co-administration of rutin (50 mg/kg, orally). BPA exposure decreased SOD activity, while DBP and DBP + BPA reduced catalase (CAT) activity and downregulated glutathione (GSH) and nuclear factor erythroid 2–related factor 2 (Nrf2) levels. MDA and CRP levels increased in the DBP + BPA group, along with elevated nuclear factor kappa B (NF-κB) expression. Rutin supplementation effectively restored SOD, GSH, and Nrf2 levels, while reducing MDA, CRP, and NF-κB expression. These findings indicate that BPA and DBP induce cardiac oxidative stress and inflammation via the Nrf2/NF-κB signaling pathway, and that rutin mitigates these effects by upregulating Nrf2 and suppressing NF-κB activation [54]. Notably, rutin is one of the flavonoids found in watercress extract [55].

Research has also shown the potential of a standardized (hydro-glycolic extract) extract of *Nasturtium officinale* (SENO) from leaves in reducing oxidative stress and inflammation in individuals with physical disabilities. SENO supplementation has been associated with reductions in lipid peroxidation, protein carbonyls, catalase, SOD, and CRP levels. However, it reduces cytokines to undetectable levels, limiting the assessment of inflammation [40]. SENO supplementation has also proved to significantly improve LDL cholesterol, creatinine levels, and markers of lipid peroxidation [25]. These improvements are clinically relevant as elevated LDL cholesterol and oxidative stress are established contributors to the development and progression of CVDs [56,57,58].

In a separate study, supplementation of *Nasturtium officinale* ethanolic extract (EENO) from leaves to hemodialysis patients resulted in significant reductions in serum MDA and BUN levels, along with increased SOD activity. Interestingly, total antioxidant capacity (TAC) was stabilized, and rises in LDL and triglycerides (TGs) were prevented. These results suggest that EENO may enhance antioxidant defenses and support lipid control [41].

Some researchers reported that fresh watercress leaves consumption by healthy individuals induced a mild pro-inflammatory response marked by increased IL-1β, IL-6, and TNF-α while simultaneously promoting the downregulation of IL-6 and TNF-α, suggesting a modulatory effect on the immune system [24]. These collective findings highlight the crucial roles of metabolic processes, oxidative stress, and inflammation in the progression of cardiovascular diseases, as demonstrated across numerous preclinical and clinical settings [57,59,60,61,62]. Thus, watercress shows potential benefits in the prevention and management of CVDs.

### 3.2. Effects of Watercress on Cancer

Cancer is a disease marked by the breakdown of normal regulatory mechanisms that control cell proliferation, resulting in the unchecked growth of malignant cells. This dysregulation arises from genetic and epigenetic alterations that impair critical signaling pathways governing the cell cycle, apoptosis, and differentiation [63,64]. It is the second leading cause of death among all non-communicable diseases globally [2].

Cancer cells can spread by breaking away from the original tumor, entering the bloodstream, and forming new tumors in other parts of the body. They use enzymes such as matrix metalloproteinases (MMPs) to break down surrounding tissues, create new blood vessels, and avoid the immune system (angiogenesis and immune evasion) to support their growth and spread [65,66,67]. Recent studies show that liquid–liquid-phase separation (LLPS) helps cancer cells grow and spread by controlling signals, gene activity, and metabolism. The tumor’s surroundings including support and immune cells also play a key role in helping cancer cells survive, invade, and resist treatment [64,67].

In pre-clinical and clinical trials, watercress has exhibited anticancer effects by inhibiting cancer cell growth, reducing DNA damage, and suppressing tumor development, likely due to its high content of isothiocyanates such as PEITC. It also promotes apoptosis and inhibits cancer cell invasion [6,12,13,26,29,68].

Giallourou et al. supported the anticancer role of PEITC and watercress extracts in breast cancer cells. Their study demonstrated that PEITC sensitized MCF-7 cells to ionizing radiation (IR), enhancing DNA damage and cell death, whilst protecting non-tumorigenic MCF-10A cells from IR-induced damage. These differential effects were associated with the modulation of glutathione levels and redox balance, suggesting watercress as a potential adjunct in radiotherapy [27]. Arumugam et al. reported that gluconasturtiin, a glucosinolate found in watercress, is hydrolyzed by the enzyme myrosinase to form gluconasturtiin-isothiocyanate (GNST-ITC), which significantly inhibited the proliferation of HepG2 (human hepatocarcinoma) and MCF-7 (human breast adenocarcinoma) cells with IC_50_ values of 7.83 μM and 5.02 μM, respectively. GNST-ITC induced apoptosis characterized by chromatin condensation, nuclear fragmentation, and membrane blebbing, and triggered G2/M-phase cell cycle arrest and activation of caspase-3/7 and -9, indicating a mitochondrial-mediated pathway [69].

Kyriakou et al. in 2022 compared the anticancer potential of edible (leaves and buds) and non-edible (stems) parts of watercress in melanoma (A375), non-melanoma (A431), and HaCaT cells in an in vitro model. Extracts from the edible parts exhibited higher concentrations of PEITC and other phytochemicals, greater cytotoxicity, and stronger induction of oxidative stress and apoptosis, underscoring their therapeutic potential and the value of watercress by-products [6].

Kyriakou et al. in 2023 investigated the mechanism of action of a PEITC-rich extract (PhEF) in melanoma and keratinocyte cells. PhEF selectively induced apoptosis in melanoma cells (A375 and COLO-679), with minimal effects on non-tumorigenic HaCaT cells. Early mitochondrial and endoplastic reticulum (ER) ultrastructural alterations were observed, followed by membrane depolarization and caspase activation, implicating the intrinsic apoptosis pathway modulated by Ca^2+^ efflux [13]. Furthermore, Kyriakou et al. in 2024 showed that PEITC-enriched fractions from watercress flowers induced oxidative stress and cytotoxicity in both primary (A375) and metastatic (COLO-679) melanoma cells. Cytotoxic effects were attributed not only to PEITC itself but also to its N-acetyl cysteine conjugated metabolites, indicating complex mechanisms of action [70].

Tragkola et al. evaluated a chemically characterized watercress extract against multiple melanoma cell lines (A375, COLO-679, and COLO-800), showing time- and dose-dependent cytotoxicity and activation of intrinsic apoptosis via caspase cascade. Non-melanoma (A431) and non-tumorigenic (HaCaT) cells displayed resistance, highlighting the extract’s selectivity and therapeutic relevance [28]. Yayintas et al. synthesized gold nanoparticles (AuNPs) using *Nasturtium officinale* (NO) extract, which exhibited improved apoptotic activity in A549 lung cancer cells compared to the crude extract. *NO*-AuNPs induced significant DNA damage and apoptotic cell death while being non-toxic at lower doses, indicating their promise as lung cancer therapeutics [71].

Adlravan et al. explored another innovative approach by encapsulating *NO* extract in PEG-PLGA nanoparticles (NOE-NPs), targeting A549 cells in vitro. The researchers reported that NOE-NPs demonstrated lower IC_50_ values over time and significantly increased apoptosis compared to free NOE. qPCR analysis revealed upregulation of p53, Bax, and Caspase-3, and downregulation of Bcl-2 and CyclinD1, confirming enhanced pro-apoptotic activity [68]. Taghavinia et al. extended these findings in an in vivo colorectal cancer model in mice. They compared non-encapsulated and nanoliposome-encapsulated phenolic-rich fractions (PRFs) of watercress. The nanoliposome-encapsulated PRFs showed superior regulation of apoptotic and antioxidant gene expression (e.g., Caspase-3, Bax, Bcl-2, iNOS, SOD), improved intestinal morphology, and better health outcomes, likely due to enhanced absorption, bioavailability, and bioactivity [72].

### 3.3. Effects of Watercress on Diabetes

Diabetes mellitus is a chronic metabolic disorder marked by hyperglycemia due to impaired insulin secretion, insulin action, or both. In type 1 diabetes, the autoimmune destruction of pancreatic beta cells causes absolute insulin deficiency [2,73]. In T2D, insulin resistance in peripheral tissues combines with progressive beta-cell dysfunction, leading to inadequate insulin secretion over time [74]. These disruptions in glucose metabolism lead to persistent high blood sugar and can cause systemic complications affecting the cardiovascular, renal, and nervous systems. Chronic hyperglycemia also promotes inflammation and oxidative stress, further contributing to organ damage and disease progression [75].

Several drug classes help in the management of diabetes. Alongside medication, diet is crucial, and plant metabolites like flavonoids, alkaloids, terpenes, and phenolics have shown significant potential in diabetes management [76]. Bayram et al. synthesized ZnO nanoparticles using *Nasturtium officinale* leaf extract and tested them in alloxan-induced diabetic rats. The extract-enriched ZnO nanoparticles showed superior effects in lowering blood glucose and improving insulin and lipid profiles compared to the extract alone, ZnO alone, or insulin [33]. Building on this, Chen et al. used a T2D mouse model to evaluate watercress activity. Both dried powder and water-soluble extracts improved glucose levels, lipid profiles, insulin signaling (via IRS-1, IRS-2, PI3K, AKT-2, and GLUT4), and antioxidant enzyme activity, indicating enhanced metabolic and oxidative stress regulation [77]. Rashid et al. in 2021 reported that watercress exhibited antidiabetic effects in 60 diabetic patients [42].

Thabet et al. investigated the anti-diabetic effects of watercress in 60 male albino rats with streptozotocin-induced diabetes. Rats received either 100 or 200 mg/kg/day orally of watercress aqueous extract from the aerial parts for 8 weeks. Compared to diabetic controls, treated groups showed significant reductions in fasting glucose, HbA1c, lipids (TG, TC, LDL, HDL), kidney (creatinine, urea), and liver (ALT, AST) markers, along with increased insulin levels. The 200 mg/kg dose showed the most pronounced hypoglycemic and hypolipidemic effects [30]. Yousef et al. extended these findings by demonstrating that giving 100 or 200 mg/kg/day of watercress aqueous extract to diabetic rats also upregulated key glucose metabolism genes (GLUT4 and AMPK) after 4 weeks of intervention, further supporting its role in glycemic control [31]. Kijkuokool et al. explored processing effects and found that roasting watercress preserved the highest levels of bioactive compounds and maintained strong antioxidant, antidiabetic, and α-amylase inhibitory activity, emphasizing the importance of preparation methods in preserving therapeutic properties [10].

### 3.4. Effects of Watercress on Chronic Respiratory Diseases

Chronic respiratory diseases (CRDs) encompass a group of conditions that affect the airways and other structures of the lungs, with chronic obstructive pulmonary disease (COPD) and asthma being the most prevalent. These diseases are associated with symptoms such as airflow limitation, breathlessness, and impaired gas exchange due to the involvement of persistent inflammation and structural changes in the airways [2,78]. In COPD, exposure to irritants like tobacco smoke causes chronic inflammation, airway narrowing, mucus overproduction, and destruction of alveolar walls (emphysema), resulting in progressive and largely irreversible airflow obstruction [79,80]. Asthma is characterized by episodic, reversible airway narrowing due to bronchospasm, mucus hypersecretion, and airway hyperresponsiveness, often triggered by allergens and mediated by eosinophilic inflammation [81].

Ramezani et al. demonstrated in a bleomycin-induced pulmonary fibrosis rat model that ethanolic extract of watercress (500 mg/kg) reduced oxidative stress markers, lung fibrosis, and histological damage, showing effects comparable to vitamin E [34]. In 2022, Shakerinasab et al. found that watercress extract from leaves and stems (500 mg/kg) orally given to asthma-induced Wister rats for 7 days improved antioxidant enzyme activity (notably GPX) and reduced SMA-α expression, with histological improvements in lung inflammation [82]. Additionally, Rajizadeh et al. demonstrated that giving an intraperitoneal injection of quercetin (50 mg/kg) once daily for 7 days to asthmatic rats reduced the expression of key inflammatory genes (Gata3, TNF-α, and TGF-β1), increased antioxidant enzymes (CAT, GPX, and SOD), and enhanced anti-inflammatory cytokine IL-10 [83].

Researchers have also reported that a single intake of 85 g fresh watercress produced immediate antioxidant and anti-inflammatory effects in humans, suggesting its relevance for managing chronic respiratory diseases [43]. In 2024, a follow-up randomized, double-blind, placebo-controlled trial in asthma patients (60 adults with age ranging from 18 to 65 years) showed that 4 weeks of watercress hydroalcoholic extract (500 mg capsule) supplementation twice daily significantly lowered serum MDA, PCO, and NO metabolites and elevated ferric reducing antioxidant power (FRAP) levels, indicating improved oxidative status and potential clinical benefit [36].

Watercress significantly reduced oxidative stress markers (MDA, PCO, and NO metabolites) and increased total antioxidant capacity in asthmatic patients. This suggest that it improves oxidative stress status in asthmatic patients [36] and also works as a potential protective agent against environmental toxins [54].

## 4. Discussion

This review consolidates recent evidence on the potential of watercress and its bioactive compounds in the prevention and management of major NCDs, including cardiovascular diseases, diabetes, cancer, and chronic respiratory conditions. The principal findings highlight that watercress, due to its rich content of glucosinolates, isothiocyanates, flavonoids, vitamins, and minerals, exerts a range of beneficial effects, notably antioxidant, anti-inflammatory, metabolic regulatory, and chemoprotective actions [7,84,85,86]. Clinical and preclinical studies consistently demonstrate that watercress supplementation may improve lipid profiles, reduce oxidative stress, and modulate inflammatory responses in both cardiovascular and respiratory diseases. Furthermore, watercress extracts from the leaves, stems, and flowers appear to enhance insulin sensitivity and glycemic control in diabetes and show antiproliferative and pro-apoptotic effects in cancer models, with selective toxicity towards cancer cells and protective effects on normal cells [7,10,12,19,23,24,35].

Recent clinical trials and mechanistic studies have collectively supported the therapeutic potential of watercress as a dietary adjunct in NCDs prevention. Watercress has demonstrated impressive heart-protective benefits by lowering LDLs and triglycerides as well as reducing markers of oxidative stress like MDA and CRP after supplementation. For example, the flavonoid rutin, which is plentiful in watercress, has been found to improve endothelial function by reducing oxidative stress and inflammation caused by BPA and DBP in preclinical studies. Plus, when participants took standardized extracts (SENO, 750 mg/kg/day) it helped to regulate the Nrf2/NF-κB pathway, bringing back a healthy balance in those who are overweight or on hemodialysis. These results highlight that watercress helps to boost vascular health by working in harmony with lipid metabolism and inflammatory processes [25,40,57,60,61].

The potential of watercress as an anticancer agent is mainly linked to PEITC, a compound formed from gluconasturtiin. This substance has a unique ability to trigger cell death in cancer cells by disrupting their mitochondria functionality and activating caspases. Interestingly, extracts rich in PEITC have shown to be toxic to melanoma and breast cancer cells while leaving healthy cells unharmed, which emphasizes its targeted action against tumors. Additionally, cutting-edge delivery methods, like nanoliposome encapsulated phenolic fractions, have improved the absorption and gene regulating effects (up regulation of Bax and Caspase-3) in colorectal cancer studies [6,13,26,68,72]. These findings highlight the promise of watercress as complementary treatment alongside traditional therapies in the management of cancer.

In managing diabetes, watercress extracts have shown to be effective by enhancing glycemic control through the stimulation of beta cells found in the islets of Langerhans within the pancreas to produce insulin and the activation of insulin sensitive pathways like GLUT4 and AMPK, while inhibiting α-amylase activity. Studies on rodents revealed that these extracts led to significant reductions in fasting glucose and HbA1c levels, along with improvements in liver and kidney health markers [30,31,76,77]. In chronic respiratory diseases management, watercress has been effective in reducing oxidative damage, such as lung fibrosis in models of asthma, lowering levels of MDA and nitric oxide metabolites, and increasing antioxidant capacity, as measured by FRAP. Quercetin, an important component of watercress, has been found to decrease pro-inflammatory cytokines such as TNF-α and TGF-β1 [36,82,83,87]. However, the temporary increase in pro-inflammatory responses after consumption suggests that more research is needed to fully understand its effect on the immune system modulation.

In terms of safety when using watercress, no toxicities or side effects have been documented so far. Clemente et al. investigated the safety of a hydro-glycolic watercress extract (containing 5.0 mg/mL of phenylethyl glucosinolate) by conducting acute and sub-acute oral toxicity tests on Wistar rats. The results showed that even at acute doses of up to 5000 mg/kg there were no signs of toxicity. In the sub-acute phase, when rats were given daily doses ranging from 250 to 1000 mg/kg over four weeks, there were no negative effects on blood markers, organ function, or size [88]. In follow-up clinical trials, subjects were given 750 mg/kg/day of the extract for five weeks. Remarkably, there were no side effects, nor any signs of nephrotoxicity or hepatotoxicity observed [25,40].

When compared to the broader literature, the findings of this review are consistent with previous reports on the health benefits of cruciferous vegetables and their phytochemicals in chronic disease prevention [4,12,19,23,35]. These findings are significant for public health and nutrition. Watercress, as a nutrient-dense vegetable, represents a promising functional food that can complement existing dietary strategies for chronic disease prevention and management. Its rich phytochemical content may offer synergistic benefits when included as part of a diverse, plant-based diet.

Nevertheless, several limitations must be acknowledged. Many of the clinical studies included in this review have relatively small sample sizes, short intervention durations, and often focus on surrogate biomarkers rather than definitive health outcomes. The heterogeneity in study designs, extract preparations, and dosing regimens complicates direct comparisons and limits the generalizability of findings. Additionally, much of the mechanistic evidence is derived from preclinical models, which may not fully translate to human physiology. The potential for interactions with medications or underlying health conditions also remains insufficiently explored. Hence, robust clinical research is needed to establish definitive recommendations for its use in public health and clinical practice. Although this review used the scale for the assessment of narrative review articles (SANRA) in assessing their quality, it is still prone to selection and interpretation bias.

Future research should prioritize large-scale, long-term randomized controlled trials to confirm the efficacy and safety of watercress supplementation in diverse populations. Standardization of extract formulations and dosing regimens will be essential to facilitate comparisons across studies. Further investigations into bioavailability, metabolism, and gut microbiome interactions of watercress phytochemicals could provide deeper insight into their mechanisms of action. Additionally, research exploring the role of watercress in combination with other dietary interventions may reveal beneficial effects for NCDs prevention and management.

## 5. Materials and Methods

This review adopts a narrative approach to summarize the therapeutic potential of watercress. Relevant articles were identified using electronic databases including PubMed (http://www.ncbi.nlm.nih.gov/pubmed), Google Scholar (http://scholar.google.com), Scopus (http://www.scopus.com), and Science Direct (http://www.sciencedirect.com) accessed on 8 April 2025. In this review, authors looked at the effects of watercress on the four main NCDs (cardiovascular diseases, diabetes, cancer, and chronic respiratory diseases). The review contains 88 publications, of which 74 are original articles, 10 are review articles, 2 are conference abstracts/proceedings, and 2 are web pages, all dated from 2019 to March 2025.

Search terms included (“watercress”, OR “*Nasturtium officinale*”, OR “*N. officinale*”) OR (“*Nasturtium officinale*” OR “Watercress” AND “cardiovascular diseases” OR “diabetes” OR “cancer” OR “chronic respiratory diseases”). Both preclinical and clinical studies conducted on cell lines, animal species, and human trials published in English were considered. Studies focusing on health benefits in humans or mammalian models were prioritized. Reference lists of selected papers were also screened for additional relevant literature.

## 6. Conclusions

Current evidence highlights watercress as a promising functional food with a lot of benefits in the context of noncommunicable diseases. It has a rich composition of glucosinolates, isothiocyanates, flavonoids, and essential nutrients which exhibit antioxidants, anti-inflammatory, and metabolic effects that may reduce the risk and progression of NCDs. Clinical and preclinical studies demonstrate improvements in lipid metabolism, oxidative stress, and inflammatory biomarkers, as well as direct anticancer activities such as cell cycle arrest and the induction of apoptosis. While these findings are encouraging, further large-scale, long-term clinical trials are necessary to confirm the efficacy and safety of watercress supplementation in diverse populations and to clarify optimal intake levels. Integrating watercress into dietary strategies may offer an accessible and natural approach to mitigating the global burden of NCDs.

## Figures and Tables

**Table 1:** Phytochemical composition of watercress.

Phytochemical Group	Key Compounds Identified	Reference
Glucosinolates	Gluconasturtiin, PEITC, and MEITCs	[4,5,6,7,8,9,10]
Phenolic acids	Coumaric acid, caffeic acid, ferulic acids, chlorogenic acids, protocatechuic acid, and dicaffeoyltartaric acid	[9,10,11,12,13,14,15]
Flavonoids	Quercetin, rutin, kaempferol, and isorhamnetin	[6,7,11]
Tannins	Gallo-tannins, ellagitannins, and proanthocyanidins	[10,11,12]
Carotenoids	Beta-carotene, lutein, and zeaxanthin	[16]
Vitamins and minerals	B3, E, K, C, A, calcium, iron, magnesium, phosphorus, and copper	[5,6]
Terpenes	α-terpinolene, limonene, caryophyllene oxide, and p-cymene-8-ol	[4]

Note: PEITC: phenethyl isothiocyanate; MEITCs: methyl isothiocyanates.

**Table 2:** Recent clinical trials on the effects of watercress on NCDs which were published between 2019 and March 2025.

Study Design	Population	Intervention	Key Findings	References
RCT, double-blind, placebo-controlled	65 participants (disabled + controls)	750 mg/kg/day watercress extract (SENO) from leaves for 5 weeks	↓ Oxidative stress (lipid peroxidation and protein carbonyls) and ↓ CRP	[41]
RCT, double-blind, placebo-controlled	34 overweight participants	750 mg/kg/day (SENO) from leaves for 5 weeks	Improved LDL cholesterol, creatinine, and lipid peroxidation markers	[26]
RCT, double-blind	46 hemodialysis patients	500 mg/day ethanolic extract (EENO) from leaves for 4 weeks	↓ MDA and BUN, ↑ SOD activity, and stabilized LDL/TG levels	[42]
Crossover intervention study	19 healthy subjects (14 males and 5 females)	Fresh watercress leaves consumption	Immune modulation: ↓ IL-6/TNF-α and mild pro-inflammatory response (↑ IL-1β/IL-6)	[25]
Crossover acute intervention study	4 healthy adults (1 male and 3 females)	Single dose (85 g fresh watercress leaves)	Acute antioxidant and anti-inflammatory effects	[44]
Non-randomized clinical trial	60 diabetic patients	Watercress leaf extract (20 mL/day orally) for 6 weeks	Reduced blood glucose levels	[43]
RCT, double-blind, placebo-controlled	Asthma patients	500 mg watercress leaf extract twice daily for 4 weeks	↓ Oxidative stress (MDA and PCO) and ↑ total antioxidant capacity (FRAP)	[37]

Note: ↓: decreased; ↑: increased; RCT: randomized control trial; SENO: standardized extract of *Nasturtium officinale*; EENO: ethanolic extract of *Nasturtium officinale*; CRP: C-reactive protein; LDL: low-density lipoprotein; MDA: malondialdehyde; BUN: blood urea nitrogen; SOD: superoxide dismutase; TG: triglyceride; IL-1β: interleukin-1 beta; IL-6: interleukin-6; TNF-α: tumor necrosis factor-alpha; PCO: protein carbonyls; FRAP: ferric reducing antioxidant power.

## Data Availability

The data of the manuscript is within the paper.

## Abbreviations

The following abbreviations are used in this manuscript:

NCDs	Non-Communicable Diseases
CVDs	Cardiovascular Diseases
CRDs	Chronic Respiratory Diseases
PEITC	Phenethyl Isothiocyanate
LDL	Low-Density Lipoprotein
RCT	Randomized Controlled Trial
SENO	Standardized Extract of *Nasturtium officinale*
CRP	C-Reactive Protein
EENO	Ethanolic Extract of *Nasturtium officinale*
MDA	Malondialdehyde
BUN	Blood Urea Nitrogen
SOD	Superoxide Dismutase
TGs	Triglycerides
T2D	Type 2 Diabetes
AGEs	Advanced Glycation End-Products
RAGEs	Receptor for Advanced Glycation End-Products
IRS1	Insulin Receptor Substrate 1
PI3K	Phosphoinositide 3-Kinase
AKT2	Protein Kinase B (AKT2) pathway
BPA	Bisphenol A
DBP	Dibutyl Phthalate
CAT	Catalase
GSH	Glutathione
Nrf2	Nuclear Factor Erythroid 2–Related Factor 2
NF-κB	Nuclear Factor Kappa B
IL	Interleukin (e.g., IL-1β, IL-6)
TNF-α	Tumor Necrosis Factor-alpha
AP-1	Activator Protein 1
CCL2	Chemokine (C-C motif) Ligand 2
TMAO	Trimethylamine N-oxide
WNT/β-catenin	WNT Signaling Pathway/Beta-catenin
ER	Endoplasmic Reticulum
IC_50_	Half Maximal Inhibitory Concentration
PRF	Phenolic-Rich Fraction
PhEF	Phenethyl Isothiocyanate-Enriched Extract
AuNPs	Gold Nanoparticles
PEG-PLGA	Polyethylene Glycol–Polylactic-co-Glycolic Acid
NOE-NPs	*Nasturtium officinale*-Extract Nanoparticles
HbA1c	Hemoglobin A1c
ALT	Alanine Aminotransferase
AST	Aspartate Aminotransferase
GLUT4	Glucose Transporter Type 4
AMPK	AMP-Activated Protein Kinase
COPD	Chronic Obstructive Pulmonary Disease
GPX	Glutathione Peroxidase
SMA-α	Alpha-Smooth Muscle Actin
NO	Nitric Oxide
FRAP	Ferric Reducing Antioxidant Power
Gata3	Transcription Factor GATA-3
TGF-β1	Transforming Growth Factor Beta 1
MEITCs	Methyl Isothiocyanates
PCOs	Protein Carbonyls
